# Gut Microbiota, Probiotics and Physical Performance in Athletes and Physically Active Individuals

**DOI:** 10.3390/nu12102936

**Published:** 2020-09-25

**Authors:** Maija Marttinen, Reeta Ala-Jaakkola, Arja Laitila, Markus J. Lehtinen

**Affiliations:** DuPont Nutrition & Biosciences, Danisco Sweeteners Oy, Sokeritehtaantie 20, 02460 Kantvik, Finland; reeta.ala-jaakkola@dupont.com (R.A.-J.); arja.laitila@dupont.com (A.L.); markus.lehtinen@dupont.com (M.J.L.)

**Keywords:** gut microbiota, probiotics, athletes, exercise, physical activity, physical performance, cognitive performance, recovery

## Abstract

Among athletes, nutrition plays a key role, supporting training, performance, and post-exercise recovery. Research has primarily focused on the effects of diet in support of an athletic physique; however, the role played by intestinal microbiota has been much neglected. Emerging evidence has shown an association between the intestinal microbiota composition and physical activity, suggesting that modifications in the gut microbiota composition may contribute to physical performance of the host. Probiotics represent a potential means for beneficially influencing the gut microbiota composition/function but can also impact the overall health of the host. In this review, we provide an overview of the existing studies that have examined the reciprocal interactions between physical activity and gut microbiota. We further evaluate the clinical evidence that supports the effects of probiotics on physical performance, post-exercise recovery, and cognitive outcomes among athletes. In addition, we discuss the mechanisms of action through which probiotics affect exercise outcomes. In summary, beneficial microbes, including probiotics, may promote health in athletes and enhance physical performance and exercise capacity. Furthermore, high-quality clinical studies, with adequate power, remain necessary to uncover the roles that are played by gut microbiota populations and probiotics in physical performance and the modes of action behind their potential benefits.

## 1. Introduction

The human gastrointestinal (GI) tract harbors a vast number of microbial cells (10^14^), which surpasses the number of cells that make up the human body [1]. Although many intestinal microbiota species are beneficial, others are potentially detrimental, or their functions remain unknown. These resident microbes are involved in many metabolic processes, such as the fermentation of undigested carbohydrates into short-chain fatty acids (SCFAs), lipid metabolism, and vitamin synthesis. Intestinal microbiota also stimulates the maturation of the immune system and protects against potentially pathogenic microbes [2]. Further, the microbiota may play a role in cognitive performance and stress tolerance [3,4].

A healthy adult gut is characterized by a high degree of microbial richness (diversity) [5], favoring health-promoting species, and features an intact epithelial barrier, which affects the inflammatory status and nutrient utilization of the host [6]. Genetic and environmental factors, in addition to diet and antibiotic use, have major influences on the gut microbiota composition, starting in early childhood and extending into adulthood [7]. Dysbiosis and the loss of diversity among gut microbiota species have been associated with various immune-regulated pathological conditions and diseases and may, in part, contribute to the risks of developing obesity-related disorders [7,8]. Gut microbiota populations with high degrees of microbial diversity have been associated with various health benefits in adults. Gut microbes have the potential to exert effects via metabolites, such as SCFAs and neurotransmitters, that can influence mucosal tissues locally or enter the circulation to affect extra-intestinal tissues. Recently, these findings have resulted in the conceptualization of a gut-brain axis (for review see [9]) and a gut-muscle axis (for review see [10]) indicating the existence of bidirectional communications between the gut microbiota and the peripheral tissues of the host.

Exercise has well-known effects on cardiorespiratory fitness, muscle strength, glucose metabolism, the immune system, and mental health [11]. Emerging evidence has indicated a plausible association between physical activity and the gut microbiota composition [12,13,14]. The particular features of gut microbiota compositions found in athletic individuals and the impacts of exercise on the gut microbiota compositions of sedentary populations have begun to be revealed. Intervention studies have supported the beneficial impacts of exercise and physical activity on the gut microbiota [15,16,17]. Furthermore, a growing interest has developed regarding whether the modification of the gut microbiota composition can affect the exercise and training outcomes of the host.

Probiotics are, by definition, “live micro-organisms that, when administered in adequate amounts, confer a health benefit on the host” [18]. Probiotic supplementation may modify the gut microbiota composition, promoting increased microbial diversity and supporting the growth of health-promoting species [19,20,21]. Probiotics may also help restore a disturbed gut microbiota [15] and support a microbiota under stress [22,23]. Although, many probiotics can support a general healthy GI and immune system function, the specific mechanisms underlying probiotic actions, such as the production of bioactive compounds, the inhibition of pathogen adhesion, the improvement of gut barrier function, and immune modulation, may be highly strain-specific, even within a single bacterial species [18].

Thus far, probiotic research has primarily focused on GI function and immune regulation; however, recent studies have targeted new research areas, such as metabolic and cognitive health. The well-established probiotic effects on gut health and immune system function may benefit endurance athletes, who train and perform at high intensities and often encounter physiological challenges associated with GI and immune health during and after a competition. Therefore, probiotic supplementation may indirectly improve the performance of an athlete by increasing the number of healthy training and competition days and maybe even benefit stamina. The benefits of probiotics for sports performance and training have been recognized, although the number of studies that have examined these issues remains limited. Recently, the International Society of Sports Nutrition (ISSN) provided a position stand on probiotics, concluding that probiotics have strain-specific effects in athletes [24]. In this review, we provide an overview of the current research on the relationships between exercise and gut microbiota and further evaluate the indirect and direct effects of probiotics on physical performance, in animal models and human subjects.

## 2. Gut Microbiota and Physical Performance

Exercise has well-known effects on metabolism and the immune system, but the effects of exercise on the gut microbiota have been less well studied. Compared with sedentary subjects, athletes and physically active subjects appear to have greater fecal microbial diversity and more health-associated microbial genera, such as *Akkermansia*, *Veillonella* and *Prevotella* [12,13,14]. However, the results of these observational studies can only confirm associations between training status and microbiota populations, without determining causality. In addition to physical activity patterns, sedentary subjects often differ from physically active subjects in dietary intake patterns [25], and diet has a strong impact on the gut microbiota composition [26].

The association between exercise and the gut microbiota composition appears to be bidirectional. Exercise intervention studies in humans have indicated that regular physical activity modulates the gut microbial composition [15,16,17]. Furthermore, growing evidence from animal studies has also suggested that the gut microbiota plays an important role in the physical performance of the host [27,28,29]. The composition and metabolic activity of gut microbiota may aid in the digestion of dietary compounds and improve energy harvest during exercise, which could provide metabolic benefits for an athlete during high-intensity exercise and recovery. Observational studies have demonstrated that the metabolic activity and pathways associated with amino acid and carbohydrate metabolism are increased among the athlete microbiome compared with those in sedentary subjects [13,14,30].

In the gut, bacteria ferment non-digestible carbohydrates, primarily into SCFAs acetate, propionate and butyrate. Training and regular exercise have been associated with increased fecal SCFA contents in humans [15,30], and specific SCFAs have been associated with improved physical performance in animal studies [14,29]. Most SCFAs are absorbed from the intestinal tract and contribute to the host’s energy metabolism [31]. Butyrate is used primarily by epithelial cells in the colon, as an energy source. Acetate is metabolized in muscle tissue but can also cross the blood-brain barrier. Propionate can be used as a precursor for glucose synthesis in the liver [31]. Additionally, SCFAs improve intestinal barrier integrity, reducing local and systemic inflammation risk. Preclinical studies have strongly suggested that SCFAs may represent key modulators of physical performance.

**Figure 1:**
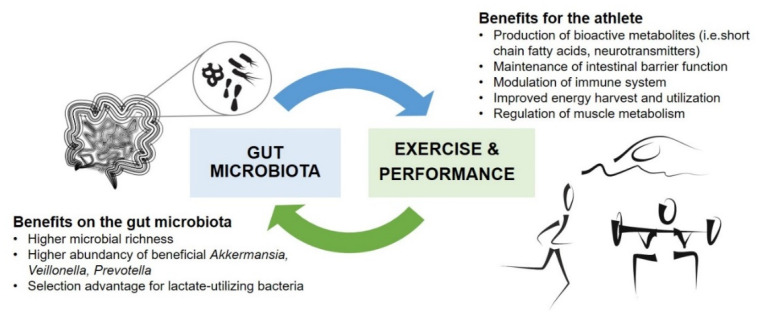
Interactions between gut microbiota and exercise.

Notably, the host may not be the only party to benefit from the symbiotic relationship with microbiota during exercise. A recent study suggested that lactate, produced by the host skeletal muscles during anaerobic exercise, enters the gut lumen through circulation, providing a selective advantage for lactate-utilizing species that reside in the colon [14]. The results from this seminal work imply that during high-intensity exercise, the host provides fuel, in the form of lactate, for specific bacteria, which, in turn, produce metabolites, such as propionate, that benefit the exercising host. Current research on the interactions between the gut microbiota and physical performance is reviewed below and summarized in Figure 1.

### 2.1. Gut Microbiota in Athletes

Accumulating clinical evidence has suggested that exercise modifies the gut microbiota and that the gut microbiota composition in athletes differs from that in sedentary people, with athletes presenting with microbial populations that are enriched in health-promoting species and have greater diversity (Table 1). Diet and specific dietary components, such as dietary fiber, have been identified as major influencers of the gut microbiota composition [26]. In cross-sectional and longitudinal studies, the impacts of diet on gut microbiota cannot be excluded, especially because the dietary intake of an athlete can greatly differ from the intake of a sedentary individual, in terms of both caloric and nutrient contents. Most of the studies in Table 1 have reported dietary intake.

A study involving Irish, male professional rugby players showed a higher α-diversity (bacterial richness, such as how many bacterial species are identified in fecal samples) for the gut microbiota of athletes compared with those in sedentary controls [12]. Gut microbiota diversity correlated positively with protein consumption and plasma creatine kinase (CK) levels, a biomarker for exercise-induced muscle damage. A higher proportion of bacteria from the *Akkermansia* genus was detected in rugby players and controls with low body mass index (BMI) compared with the proportion in controls with high BMI. *Bacteroides* spp. were significantly less abundant in athletes than in controls with low BMI.

**Table 1:** Studies on exercise and gut microbiota conducted in athletes, physically active individuals and sedentary population.

Subjects	Training Regimen, Exercise Protocol	Dietary Intake	Main Results	Reference
**Athletes:**				
Rugby players vs. BMI-matched sedentary controls n = 86, males Age 29 ± 4 y	Habitual training and exercise	Self-reported intake by FFQ In athletes, higher total energy, macronutrient and fiber intake. Protein intake 22 E% in athletes, 16 E% in low-BMI and 15 E% in high-BMI controls	In athletes, higher α-diversity and *Akkermansia* spp. abundance vs. sedentary controls. Protein intake was positively correlated with microbial diversity.	[12]
Rugby players vs. BMI-matched sedentary controls n = 86, males Age 29 ± 4 y	Habitual training and exercise	Self-reported intake by FFQ In athletes, higher total energy, macronutrient and fiber intake. Protein 22 E% in athletes vs. 16 E% in low-BMI and 15 E% in high-BMI controls	In athletes, fecal SCFAs, microbial pathways for antibiotic biosynthesis, and amino acids and carbohydrate metabolism were increased.	[30]
Professional cyclists vs. amateur cyclists n = 33 (22/M, 11/F) Age 19–49 y	Habitual training	Dietary intake data collected by questionnaire, reported and analyzed as overall dietary patterns.	*Prevotella* spp. abundance was positively correlated with the amount of exercise and branched chain amino acid and carbohydrate metabolism pathways. Professional cyclists had increased *Methanobrevibacter smithii* transcripts and upregulated genes involved in the production of methane compared with amateur cyclists. No correlations between overall diet and gut microbiota clusters.	[13]
Cross-country runners n = 18, males Age: Control group 35.4 ± 9.0 y Protein group 34.9 ± 9.5 y	Habitual endurance training	Habitual diet by FFQ No differences in habitual dietary intake within or between groups, at baseline or after the intervention. Dietary intervention: habitual diet and whey isolate (10 g) + beef hydrolysate (10 g) or maltodextrin (control) for 10 weeks	After the intervention, higher Bacteroidetes and lower Firmicutes abundance in the protein group. *Bifidobacterium longum* was reduced after intervention in the protein group. No changes in microbiota composition in the control group, from pre- to post-intervention. No differences within or between groups in fecal SCFA, before or after the intervention.	[32]
Bodybuilders, long-distance runners vs. sedentary subjects n = 45, males Age: Bodybuilders 25 ± 3 y, distance runners 20 ± 1 y, sedentary 26 ± 2 y	Habitual training and exercise	Self-recorded 3-day food diary Bodybuilders had a high-protein and distance runners had a low-dietary-fiber dietary pattern. Dietary fiber intake was below recommendation in all groups.	Compositional differences in bodybuilders and runners associated with exercise type and diet. No difference in microbial diversity between groups. In distance runners, protein intake was negatively correlated with microbial diversity.	[33]
Highly trained ultra-endurance rowers n = 4, males Age 26.5 ± 1.3 y	ca. 5000 km rowing race over 34 days	Self-reported intake (FFQ), detailed daily record pre-race and during the race No fresh produce consumed during race. Pre-race fiber intake: 21.45 g/day, intra-race 23.1 g/day. Only small changes in intra-race macronutrient intake compared with pre-race	After the race, increased diversity and butyrate-producing species including *Roseburia hominis* and changes in microbial composition were observed.	[34]
Elite race walkers n = 21, males Age 20–35 y	3-week structured program of intensified training	Dietary intervention for 3 weeks with planned and individualized menus. Subjects allocated into High-carbohydrate diet (HCHO) Periodized-carbohydrate diet (PCHO), or Low-carbohydrate, high-fat diet (LCHF) (ketogenic) group	At baseline, microbiota profiles could be separated into *Prevotella-* or *Bacteroides*-dominating enterotypes. HCHO and PCHO resulted in minor changes, whereas LCHF resulted in stronger changes in microbial composition. LCHF was associated with reduced *Faecalibacterium*, *Bifidobacterium,* and *Veillonella* spp. Increased *Bacteroides* and *Dorea* spp. in the LCHF group was associated with decreased performance.	[35]
Marathon runners: n = 15 (4/M, 11/F) Mean age 27.1 y; Non-runners: n = 11 (5/M, 6/F) Mean age 29.2 y; Ultramarathon and rower athletes: n = 11 (5/M, 6/F) Age not reported	Habitual training and a marathon Type of exercise not reported for the cohort of ultra-marathon and rower athletes	Dietary intake data collected by questionnaire	In marathon runners, the relative abundance of *Veillonella* spp. increased post-marathon. In ultramarathon and rower athletes, the relative abundance of the methylmalonyl-CoA pathway (degrading lactate into propionate) in the gut microbiome increased post-exercise. No correlations between dairy, protein, grains, fruits, or vegetables and *Veillonella* spp. abundance was observed among marathon runners.	[14]
**Non-athletes and sedentary subjects:**				
Healthy subjects n = 39 (22/M, 17/F) Age 18–35 y	VO_2_Peak test to assess CRF and to allocate subjects into groups (low, average, and high CRF)	24-h dietary recall interview No significant differences in dietary intake between groups.	CRF correlated with microbial diversity and butyrate production.	[36]
Active vs. sedentary women n = 40 Active: 30.7 ± 5.9 y, BMI 24.4 ± 4.5 kg/m^2^; Sedentary: 32.2 ± 8.7 y, BMI 22.9 ± 3.0 kg/m^2^	Habitual physical activity measured by accelerometer.	Self-reported food intake (FFQ) Fiber, fruit, and vegetable intake significantly higher in the active group.	Higher abundance of *Faecalibacterium prausnitzii, Roseburia hominis* and *Akkermansia muciniphila* in active women. Physical activity was not associated with differences in microbiota richness.	[37]
Lean and obese sedentary subjects n = 32 Lean: n = 18 (9/M, 9/F), mean age 25.10 y; Obese: n = 14 (3/M, 11/F), mean age 31.14 y	Exercise intervention study: 6 weeks of moderate-to-vigorous intensity aerobic exercise and 6 weeks without exercise	Maintenance of habitual diet during the intervention. A designed 3-day food menu, based on previous reported habitual diet, before fecal sample collection.	At baseline, the composition of gut microbiota differed between lean and obese subjects, but after exercise training, no difference was observed between lean and obese subjects. Exercise increased fecal SCFA and SCFA producing bacteria in lean subjects.	[15]
Children and teenagers n = 267 (178/M, 89/F) Age 7–18 y	Self-reported physical activity	Type of diet reported as omnivore or vegetarian.	Gut microbiota composition was affected by BMI, exercise frequency, and diet type. Firmicutes were significantly enriched in subjects with more frequent exercise.	[38]
Overweight sedentary women n = 17 Age 36.8 ± 3.9 y BMI 31.8 ± 4.4 kg/m^2^	Habitual physical activity. Exercise intervention study: 6-week control period without exercise, 6-week programmed endurance exercise, on a bicycle ergometer	Habitual diet Self-reported 3-day food record No changes in intake of total energy, macronutrients or fiber from baseline, after control or exercise period. A modest increase in energy from starch	Exercise did not affect α-diversity. Exercise increased *Akkermansia* spp. and reduced Proteobacteria abundance. No significant changes in BMI or total fat mass after exercise. Significant reduction in android fat mass.	[16]
Healthy subjects n = 37 (20/M, 17/F) Age 25.7 ± 2.2 y	VO_2max_ test to assess CRF	Habitual diet recorded for 7 days	CRF correlated with Firmicutes/Bacteroidetes ratio. No correlation between dietary factors or BMI and Firmicutes/Bacteroidetes ratio.	[39]
Elderly community-dwelling men n = 373 Age 78–98 y	Habitual physical activity, measured by activity sensor, for 5 days. Step count as primary physical activity variable	Self-reported food intake (FFQ) Step count was not associated with food or alcohol intake.	Physical activity was not associated with α-diversity but was positively associated with β-diversity. Increased physical activity was associated with greater *Faecalibacterium* and *Lachnospira* spp. prevalence.	[40]
Elderly sedentary women n = 29 Age 65–77 y	Exercise intervention study: resistance training (trunk muscles) or aerobic exercise (brisk walking) for 12 weeks	Self-reported food intake (FFQ) No changes in energy or nutrient intake after interventions.	Brisk walking increased the relative abundance of *Bacteroides* spp. *Bacteroides* spp. abundance was positively associated with improved CRF after aerobic training but not with improved CRF after resistance training.	[17]

BMI, body mass index; y, years; FFQ, food frequency questionnaire; E%, percentage of total energy intake; SCFA, short-chain fatty acid; M, males; F, females; VO_2Peak_/VO_2Max_, maximum rate of oxygen consumption; CRF, cardiorespiratory fitness.

*Akkermansia* sp. has been shown to inversely correlate with obesity [41] and *Bacteroides* spp. has been associated with a “Western” type of diet, with high protein and fat contents [42].

Differences between rugby players and sedentary controls were also detected in the microbial metabolism level, with increased amino acid and carbohydrate metabolism pathway activity detected in athletes [30]. Furthermore, higher fecal SCFA (acetate, propionate, and butyrate) levels were detected in rugby players compared with those in sedentary controls. SCFAs produced by gut bacteria have well-known health-promoting effects on the maintenance of intestinal barrier function, immune modulation, and the host’s energy metabolism [43,44].

Similar to Clarke et al. [12], Petersen et al. [13] reported lower levels of *Bacteroides* spp. in competitive cyclists. Cyclists who trained >11 h/week had a higher relative abundance of *Prevotella* spp. than those who trained less often. In addition, a meta-transcriptomics analysis showed that *Prevotella* transcripts were positively correlated with branched-chain amino acid (BCAA) metabolism pathways in the microbiome. BCAAs, especially leucine, are essential amino acids that promote muscle protein synthesis and may enhance recovery after exercise. Further, more fecal *Methanobrevibacter smithii* transcripts were identified in professional cyclists compared with amateur cyclists. *M. smithii* was associated with upregulated methane metabolism, which correlated positively with upregulation of SCFA metabolism pathways in the gut microbiome [13]. However, the authors recognized the lack of dietary control and the absence of a non-athlete control group in the study. In line with the results observed in cyclists, fecal microbiotas were classified into *Prevotella-* or *Bacteroides*-dominant enterotypes in a small group of elite race walkers [35].

Scheiman et al. [14] demonstrated that the relative abundance of *Veillonella* spp. bacteria among marathon runners was significantly higher after the marathon, compared with the pre-exercise abundance. In addition, the same research group conducted metagenomic analyses using fecal samples from ultramarathoners and Olympic level rowers, which revealed the enrichment of genes associated with lactate and propionate metabolism in post-exercise compared with pre-exercise samples. A follow-up study, conducted in mice, demonstrated that treatment with a *Veillonella* sp. strain, which was isolated from a marathon runner, increased the treadmill running time of mice by 13% [14].

The chronological impact of prolonged, very-high-intensity exercise on the gut microbial composition was investigated in four well-trained men who participated in a trans-oceanic rowing competition [34]. All, except one rower, who required antibiotic treatment before mid-race, showed increased microbial α-diversity at mid-race, which continued until the end of the race. Baseline diversity was partially or completely restored three months after the competition. Although this study represents a very small sample size, the microbial metabolic pathways related to specific amino acids and medium and long-chain fatty acids tended to increase [34]. However, the diet differed considerably during the rowing race compared with the pre-race diet; therefore, dietary change may have also contributed to the microbial diversity findings.

In addition to the high-intense training that is practiced by professional or competitive athletes, exercise that is performed at the recommended minimum level, based on the World Health Organization (WHO) guidelines of 150 min of moderate-intensity exercise each week [45], appears to sufficiently modify the gut microbiota composition [37]. Premenopausal women who practiced continuous exercise at a low dose demonstrated increased abundance of *Akkermansia muciniphila*, *Faecalibacterium prausnitzii*, and *Roseburia hominis,* compared with those in sedentary women [37]. These all are bacterial species that are associated with health-promoting and anti-inflammatory effects [43]. Moreover, *Faecalibacterium* spp. and *Roseburia* spp. are among the most abundant butyrate-producers in the human gut [43,44]. Different dietary patterns between physically active and sedentary groups may have influenced the gut microbiota composition, as the intake of dietary fiber was significantly higher in active women compared with sedentary women (mean intake 30.9 g vs. 21.4 g), and the intake of processed meat was significantly higher in the sedentary group [37].

Associations between physical activity levels and gut microbiota compositions have also been demonstrated in children [38] and seniors [40]. In a study cohort of children, aged 7–18 years, from the American Gut Project, BMI, exercise frequency, and type of diet were individually associated with the gut microbiota composition, after controlling for covariates (age, gender, and the use of antibiotics and probiotics) [38]. Exercise frequency was associated with gut microbiota enriched with Firmicutes phylum. Furthermore, children who exercised daily showed an increase in genera within Clostridiales, Lachnospiraceae, and Erysipelotrichaceae. In older men, physical activity, measured based on step count and self-reported activity, was not associated with microbial α-diversity, but modest associations between physical activity level and *Faecalibacterium* spp. and *Lachnospira* spp. were found [40].

These studies indicated the existence of differences in the gut microbiota composition between athletes or physically active populations and sedentary populations. However, some of the characteristics of the microbiota composition in athletes and physically active people may be explained by diet, rather than the effects of exercise. Athletes often follow strict diets that support training and performance, and exercise extremes are often associated with dietary extremes [12]. Protein supplements are often consumed to meet the higher protein requirements of training individuals, although the popularity of protein supplements is likely also influenced by claims regarding increased muscle mass and improved performance and recovery [46]. Thus, protein intake can be substantially higher among athletes compared with the normal population. Following high protein intake, unabsorbed protein enters the colon and promotes the growth and selection of specific bacteria. Protein supplementation (whey isolate and beef hydrolysate) for 10 weeks increased the abundance of Bacteroidetes and decreased health-related taxa, including *Roseburia* spp., *Blautia* spp., and *Bifidobacterium longum*, in runners [32]. However, the long-term effects of such alterations in the gut microbiota composition on host health remain unclear.

Differences in dietary intake between study populations may explain some of the inconsistencies observed among the results of different studies. In a clinical study in Korea, total protein intake was inversely correlated with microbial diversity [33], whereas high protein intake was associated with increased microbial diversity among Irish professional rugby players [12]. Korean athletes did not meet the dietary recommendations for dietary fiber intake (recommendation ≥ 25 g/day; median intake in bodybuilders 19 g/day, endurance athletes 17 g/day), whereas Irish rugby players had fiber intake values at the recommended level (median intake 39 g/day). Undigested dietary fiber is an important energy and carbon source for the gut microbiota, acting as a substrate for SCFA synthesis, and representing a key contributor to microbial diversity. A high-protein diet, in combination with low-dietary-fiber diet, may be harmful for the gut microbiota composition, rather than high protein intake alone [47].

Limited data, derived primarily from animal studies, have suggested that popular sports nutrition supplements, such as caffeine, BCAAs, sodium bicarbonate, and carnitine, can modify the gut microbiota composition [48]. The effects of sports nutrition supplements on the gut microbiota remain understudied among athletes.

To summarize, exercise and training have been associated with compositional changes in the gut microbiota, including increased microbial diversity and increased abundance of health-promoting microbial species. Results from large study cohorts with recreationally active subjects suggest that exercise is associated with increases in genera within Clostridiales and Lachnospiraceae [38,40]. Although several studies have investigated small populations that likely lack sufficient statistical power, it is intriguing that they commonly identify genera such as *Akkermansia* [12,37] and *Prevotella* [12,13] at higher abundance in athletes and physically active subjects. However, because the number of clinical studies remains limited, with highly different participant demographics and dietary intake—dietary fiber intake in specific—conclusions should be drawn carefully. Observational studies that have compared trained athletes and physically active subjects with sedentary subjects have suggested long-term effects of exercise training on gut microbiota composition, wherein the diet plays an important role. Sedentary and physically active subjects differ not only in their exercise patterns but also in their dietary intake and body composition, which are both factors that are associated with the gut microbiota composition.

### 2.2. Impacts of Exercise Interventions on Gut Microbiota

Because athletes often adhere to special diets that may influence the gut microbiota, exercise intervention studies can provide a more diet-independent approach for examining whether exercise has an impact on the host gut microbiota (Table 1). A research group demonstrated that exercise training intervention modified the gut microbiota composition of sedentary, non-trained, Finnish women, without changes in dietary habits, weight, or body composition [16]. The authors demonstrated that endurance exercise altered the gut microbiome of overweight, sedentary women, who participated in an exercise intervention that consisted of performing a bicycle ergometer routine, three times a week, for six weeks. The study showed no differences in total energy intake or the intake of macronutrients or dietary fiber after the training intervention. Differences were not found in the gut microbiota α-diversity or phylum-level abundance between pre- and post-intervention samples; however, endurance exercise increased relative abundance of members of the genera *Verrucomicrobia* and *Akkermansia* and decreased the number of inflammation-associated Proteobacteria in the gut. Changes in *Akkermansia* spp. and genera and species within phyla Proteobacteria and Verrucomicrobia were responsive to exercise and were independent of age, weight, percent body fat, and food intake. Another study, performed by Morita et al. [17] found that a 12-week aerobic exercise training program significantly increased the relative abundance of *Bacteroides* spp. in elderly, sedentary women, without changes in nutrient intake.

A study by Allen et al. [15] supported these findings, showing that aerobic exercise induced changes in the gut microbiota composition, independent of dietary intake, among sedentary subjects; however, BMI may influence the response of gut microbiota to exercise. In their study, obese and lean individuals had different gut microbiota compositions at baseline, but after a 6-week aerobic exercise training program, no difference was found in microbiota community composition between obese and lean. In addition, aerobic exercise increased fecal SCFA concentrations and SCFA production capacity in lean subjects. The effects of exercise on gut microbiota were reversed after training was discontinued.

Overall, aerobic exercise training improves cardiorespiratory fitness (CRF), an effect that has been demonstrated in studies by Munukka et al. [16], Allen et al. [15] and Morita et al. [17]. CRF, which was measured as the maximum rate of oxygen consumption (VO_2max_), has been observed to correlate with gut microbial diversity, fecal butyrate levels [36], and the Firmicutes-Bacteroidetes ratio [39]. The ratio between Firmicutes and Bacteroidetes phyla has been reported to be associated with body composition, with a higher fraction of Bacteroidetes associated with higher proportions of lean body mass, whereas lower levels have been associated with obesity [49].

In addition to human clinical studies, preclinical research in animal models has demonstrated that exercise changes the gut microbiota composition [50,51,52,53,54,55] and fecal SCFA concentrations, by increasing the production of butyrate [50,54], in particular. However, forced exercise, under stressful conditions, such as the exhaustive swimming test, may impact gut microbiota differently than voluntary activity, such as wheel running. In an overtraining mouse model, the gut microbial diversity was reduced in mice forced to swim to exhaustion compared with that in non-swimming mice [56].

### 2.3. Effects of Targeted Gut Microbiota Modulation on Physical Performance

Due to nutritional, genetic, and environmental factors, dissecting the exact role played by gut microbiota on exercise performance in human clinical studies can be difficult. Germ-free animal models overcome many of those challenges and have been used to demonstrate the roles played by gut microbiota on physical performance outcomes. Hsu et al. [27] studied the swimming capacities of specific pathogen-free (SPF), germ-free (GF), and *Bacteroides fragilis* gnotobiotic mice. The swim-to-exhaustion time was the shortest for GF mice and the longest for SPF mice, indicating decreased performance in the absence of gut microbiota. Similar findings regarding the reduced performance of GF mice compared with that in gnotobiotic and SPF mice were observed by Huang et al. [57].

In contrast to the above, Lahiri et al. [58] showed that GF mice and SPF mice did not differ in physical performance when exercising until exhaustion. However, GF mice demonstrated reduced muscle mass, fewer muscle fibers, and reduced muscle strength compared with SPF mice. Muscle atrophy in GF mice was associated with dysregulated mitochondrial biogenesis and reduced oxidative capacity. The transplantation of gut microbiota from SPF mice restored the muscle mass in GF mice, and treatment with a blend of SCFAs increased skeletal muscle mass and muscle strength in GF mice compared with those in untreated GF mice [58].

Antibiotic treatment drastically alters the composition of gut microbiota. Nay et al. [28] demonstrated that gut microbiota depletion, following a broad-spectrum antibiotic treatment, reduced the endurance running time of mice, and the endurance capacity was normalized after microbiota restoration through reseeding. Changes in endurance capacity were not related to changes in muscle mass, muscle fiber typology, or mitochondrial function but were associated with changes in muscle glycogen levels, which were restored after reseeding. Okamoto et al. [29] reported similar findings, in which the treadmill running time was shorter in mice treated with multiple antibiotics compared with that in non-treated controls. Okamoto et al. [29] also investigated the effects of SCFA production and its role on exercise performance, by feeding mice with fibers with differential substrate availability for microbial SCFA production in the gut. Mice fed with reduced fermentable fibers showed significantly shorter running times compared with mice fed with highly fermentable fibers, suggesting that microbiota and its substrates are both associated with physical performance. To further explore the putative role of SCFAs in performance capacity, antibiotic-treated mice were administered with a subcutaneous infusion of acetate or butyrate [29]. Acetate, but not butyrate, infusion improved the antibiotic-induced deterioration in running time.

Germ-free animals are of course an extreme model and may not explain the more subtle difference observed in the microbiota of humans. Nevertheless, studies in germ-free animal models have established a cause-effect relationship between gut microbiota and physical performance. Overall, the normalization of gut microbiota dysbiosis appeared to effectively restore exercise capacity and skeletal muscle parameters in rodents [58]. In addition, differences in gut microbiota compositions or the lack of gut microbiota have been shown to modulate exercise capacity, associated with muscle structure, muscle strength, and/or energy utilization [25,28]. Thus, the host appears to benefit from microbes through improved performance. The effects of gut microbiota are at least partially mediated by the production of SCFAs, which impact the gut and can also affect peripheral target tissues, via circulation.

## 3. Probiotics as a Potential Ergogenic Aid to Enhance Physical Performance

Nutritional ergogenic aids are dietary supplements that are consumed to help an individual exercise, enhance exercise performance capacity, enhance training adaptations, and improve recovery from exercise [59]. The use of nutritional ergogenic supplements is popular among athletes and recreationally active individuals of all age groups; however, evidence that supports the efficacy of many supplements is very limited or lacking. Performance-enhancing supplements with good or strong evidence have been identified by the International Olympic Committee and include the following: caffeine, creatine, nitrate, sodium bicarbonate, and beta-alanine [60]. Even when associated with only marginal improvements in performance, safe, proven, ergogenic dietary supplements may provide competitive benefits for an athlete.

Probiotic supplementation has been demonstrated to beneficially modify and support the gut microbiota composition [19,20,21]. Probiotics comprise many bacterial species, with the most studied probiotics belonging to the genera *Lactobacillus* (and associated genera) or *Bifidobacterium*. Associations between probiotics and physical performance and plausible mechanisms underlying these actions have been addressed in animal studies, which have suggested that probiotic supplementation protects against undesirable physiological changes that may be induced by strenuous exercise. Preclinical studies have demonstrated that probiotics can improve gut barrier properties [61] and the antioxidative status [62] and attenuate inflammatory response [63,64,65] in rodents after exhaustive exercise. However, how these protective effects are associated with physical performance outcomes has not been determined.

To date, the effects of probiotic supplementation have been studied in a variety of athletic and physically active populations, examining a variety of probiotic strains. Because the number of clinical studies on the association between probiotics and physical performance remains very low, with each study generally including a small number of participants and utilizing different exercise protocols, conclusions should be drawn carefully. The training status and training history of the participants can also influence the outcomes of exercise interventions [66]. Trained athletes and untrained individuals differ in their physiological responses to exercise [66], which can result in controversial results among different study populations. The use of resistance training programs alone during exercise intervention studies can contribute to changes in body composition and skeletal muscle organization that may supersede the impacts associated with probiotic supplementation, especially among previously sedentary populations.

**Figure 2:**
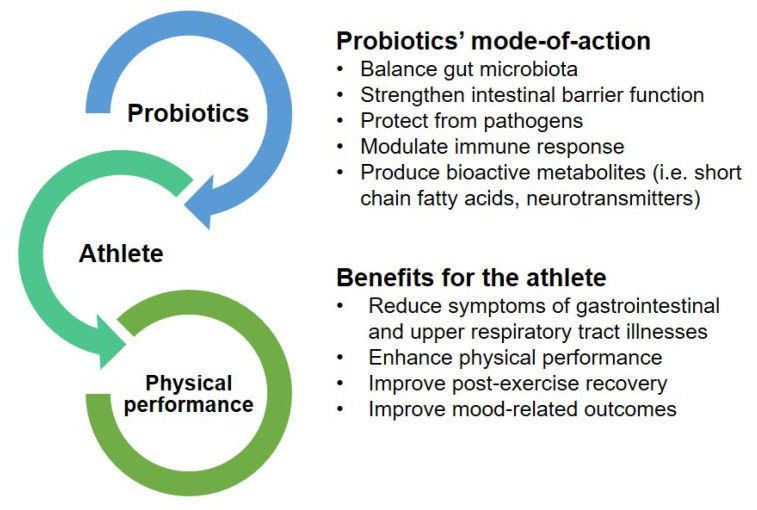
Proposed mechanisms and benefits of probiotic use in athletes.

The studies that have examined the effects of probiotics on physical performance have generally focused on mid- to long-term benefits, with supplementation periods varying from 2 weeks to 3 months (Table 2). The examined probiotic strains, formulas, and doses vary from study to study, which creates controversy among the obtained results. The most studied species are members of genera *Lactobacillus* (and associated genera) and *Bifidobacterium*; however, the benefits of probiotics, even within a single species, are often strain-specific. Furthermore, studies have been performed using both live and inactivated bacteria, which may have different modes of action. To comply with the definition, probiotics need to be alive microbes [18]. The proposed mode-of-action for probiotics and the benefits they provide to athletes are summarized in Figure 2.

### 3.1. Reduction of Gastrointestinal and Upper Respiratory Tract Symptoms

The beneficial effects of probiotics on GI health and upper respiratory tract (URT) illness symptoms among the general population have been well-acknowledged and reviewed extensively, elsewhere [67,68]. Because the exercise performance capacity of an athlete can be greatly influenced by overall health and resistance to infections, we have briefly highlighted the main findings, here.

Several studies have shown the potential of probiotic use to shorten the duration of GI disturbance episodes and relieve GI symptoms in athletes [69,70,71,72,73,74]. GI symptoms are common in athletes and can be influenced by the type of exercise performed [75]. GI challenges are most prominent in endurance athletes, among whom the prevalence of symptoms varies from 30% to up to 90%, depending on the individual athlete and the type and the extremes of the exercise [75]. Typically, endurance athletes suffer from mild to severe symptoms, including nausea, vomiting, abdominal angina, and bloody diarrhea, which are caused, in part, by reduced blood circulation in the splanchnic region during intensive exercise [75]. Reduced circulation can result in oxygen deprivation in the gut epithelial cells, which damages the cells and causes changes in the gut permeability, a phenomenon known as “leaky gut syndrome.” Metabolites, such as SCFAs, and other effector molecules, which are produced by beneficial bacteria, may improve the intestinal barrier function by increasing the expression of tight junction proteins in the epithelia, which reduces mucosal permeability [43]. Clinical results regarding the effects of probiotic administration on gut permeability in athletes are scarce and controversial, showing positive effects [72,73] or no effects [76,77].

Prolonged high-intensity exercise has been associated with transient immune dysfunction and increased illness risk [78]. Due to the transient suppression of mucosal and systemic immune responses, athletes are especially susceptible to viral respiratory infections, which affect the quality of training and physical performance [79,80]. In contrast, moderate exercise appears to protect against infections, whereas a sedentary lifestyle increases the risk [78,79]. Several studies have investigated whether probiotic supplementation can reduce the risks of respiratory tract illness episodes, alleviate symptoms, and reduce the duration of episodes among athletes and recreationally active subjects. The study results have not shown consistent effects for all of the aforementioned benefits; however, beneficial effects on incidence, duration, and the number of symptoms have been reported [69,81,82,83,84,85]. Consequently, the positive impacts of probiotic supplementation on URT symptoms and illness may facilitate an earlier return to normal activity levels, references [83,85] increasing the hours spent on training, which can positively influence overall athletic performance. The administration of a two-strain probiotic supplement (*Lactobacillus acidophilus* NCFM and *Bifidobacterium animalis* subsp*. lactis* Bi-07) delayed the occurrence of URT illness and significantly increased the training load during a 5-month intervention period compared with placebo [83].

Reducing the incidence or severity of illness has positive impacts on performance during training and competition. Thus, probiotics may indirectly enhance physical performance.

### 3.2. Enhancement of Physical Performance

Depending on the sport and exercise type, physical performance can be measured as outcomes related to endurance, strength, speed, flexibility, or psychological performance (concentration, motivation) [86]. Exercise capacity often refers to exercise time to fatigue or exhaustion, at a given intensity or workload [87]. The potential for probiotics to improve physical performance has been recognized during exercise interventions and training studies involving athletes, recreational athletes, and sedentary individuals. Table 2 summarizes the studies examining the associations between physical performance and probiotic use, including preclinical and clinical studies, in which the used supplementation protocol fulfills the definition of a probiotic as a live organism.

Probiotic supplementation has been shown to increase the time to fatigue in both preclinical studies [88,89,90] and clinical studies, among both athletes and non-athletes [77,91,92]. *Lactiplantibacillus plantarum* TWK10 is among the most studied probiotic strains in terms of physical performance outcomes. A preclinical animal study demonstrated a dose-dependent increase in forelimb grip strength and endurance swimming time in mice supplemented with TWK10 [88]. Mice supplemented with TWK10 also showed an increased number of type I (slow-twitch) muscle fibers in the gastrocnemius muscle compared with control mice. These performance benefits were further confirmed in clinical studies. Endurance performance in an exhaustive treadmill exercise was improved in healthy, untrained adult males, who were supplemented daily with TWK10 for 6 weeks, compared with those who received a placebo [91]. In addition to significantly longer time to exhaustion (58% longer running time in the probiotic vs. placebo groups), the post-exercise blood glucose level was higher in TWK10 group compared with the placebo group suggesting improved energy harvest from gluconeogenic precursors during exhaustive exercise. No significant improvements in perceived exertion during exhaustive exercise were reported by the probiotic supplementation group compared with the placebo group.

A more recent clinical study from the same research group demonstrated a dose-dependent improvement in endurance performance (time to exhaustion) following TWK10 supplementation (3 × 10^10^ or 9 × 10^10^ colony forming units, CFU, per day) in untrained subjects [92]. A higher dose of TWK10 significantly increased muscle mass compared with placebo treatment during the 6-week supplementation period. Further, blood lactate levels were significantly lower at the end of the exercise bout after both doses of probiotic supplementation compared with placebo treatment.

A double-blind, cross-over, exercise study examining trained male runners demonstrated that supplementation with a multi-strain probiotic (*L. acidophilus*, *Lacticaseibacillus rhamnosus*,* Lacticaseibacillus casei*,* L. plantarum*, *Limosilactobacillus fermentum*,* Bifidobacterium lactis*,* B. breve*,* B. bifidum*, and *Streptococcus thermophilus*) for 4 weeks significantly increased the time to fatigue on a treadmill running exercise performed in the heat compared with placebo, resulting in an average 16% longer running time [77]. No differences were observed in the severity of GI symptoms or GI permeability between the probiotic and placebo groups during exercise [77].

However, not all studies have shown enhancements in endurance performance following probiotic use in highly trained subjects or athletes [81,84,85,93]. Performance measurements related to exhaustive endurance exercise were not affected in endurance-trained males, after 4 weeks of *L. fermentum* VRI-003 supplementation [81], or in trained subjects, after *Lactobacillus helveticus* Lafti L10 [84]. A multi-species probiotic formulation (*B. bifidum* W23, *B. lactis* W51, *Enterococcus faecium* W54, *L. acidophilus* W22, *Levilactobacillus* brevis W63, and *Lactococcus lactis* W58) for 3 months did not have benefit in endurance performance in highly trained athletes [85]. In female swimmers, a multi-strain probiotic (*L. acidophilus* SPP, *L. bulgaricus*, *B. bifidum*, and *S. thermophilus*) yogurt improved the VO_2max_ (calculated using a Harvard step test) but had no impact on the 400-m swimming time after a 2-month intervention [82]. The 6-week supplementation with *B. longum* 35,624 in competitive, high-level, female swimmers did not enhance aerobic or anaerobic swimming performance or improve power or force production measurements [93]. Marshall et al. [94] found no effects for a 12-week multistrain probiotic or probiotic + glutamine supplementation protocol on the time to complete an ultra-marathon race compared with controls.

A few clinical studies have addressed the impacts of probiotic supplementation on sprint and power performance showing no clear benefits. *Bacillus subtilis* DE111 did not improve either strength or performance in male [95] or female athletes [96] when combined with a training protocol involving resistance exercises. Multi-strain probiotic supplementation for 12 weeks, combined with circuit-training, which involved resistance exercises, improved muscular performance to a similar degree as circuit-training alone in healthy, sedentary males [97], confirming the positive effects of resistance training on muscular outcomes, which is a result that has been demonstrated by other probiotic and exercise interventions among athletes [95,96]. The effects of probiotic supplementation on muscle strength and power production may be superseded by the effects of the resistance training protocols used by these studies. Regular resistance exercise strongly induces physiological changes in skeletal muscles and improves muscular strength in the long term

A recent trend in the field of gut microbiota and exercise research has been to isolate gut bacteria from the feces of elite athletes, to study the performance benefits of athlete-derived gut bacteria in animals. Recently, the oral administration of either *B. longum* subsp. *longum* OLP-01 [98] or *Ligilactobacillus salivarius* subsp. *salicinius* SA-03 [99], isolated from a female weightlifting Olympic medalist, was demonstrated to significantly increase forelimb grip strength and endurance capacity in a swim-to-exhaustion test in mice. Both OLP-01 and SA-03 significantly decreased blood lactate, ammonia, and CK levels after an acute exercise bout, and increased hepatic and muscle glycogen stores at autopsy and decreased which indicated improved energy utilization and the attenuation of fatigue-related biomarkers in mice.

The inoculation of *Veillonella atypica,* isolated from a marathon runner, increased the treadmill running time in mice compared with that of control mice [14]. In a subsequent experiment, the intracolonic infusion of propionate also improved running times until exhaustion in mice. *Veillonella* species are known to metabolize lactate into propionate and acetate. Notably, a series of experiments by this research group also showed that ^13^C_3_-lactate injected into the mouse tail vein could be found in the contents of colon and cecum, post-injection, indicating that circulating lactate can pass through the intestinal epithelium into the gut lumen. This seminal work in mice implies that lactate that is produced by skeletal muscles during prolonged anaerobic exercise may enter the colon from the circulation, which can serve as fuel for certain bacteria in the gut, providing a selection advantage [14]. These findings suggested that both the host and gut microbes may benefit from a symbiotic relationship; however, clinical evidence remains necessary to provide additional proof of these beneficial effects. Although athlete-originating microbes, such as *Veillonella,* sp. may show benefits in preclinical settings, the development of clinically proven commercial probiotics that can provide health benefits in humans requires further research. 

**Table 2:** Probiotic studies on physical performance, post-exercise recovery and cognitive outcomes.

Subjects	Design	Exercise Protocol and/or Intervention	Probiotic Supplementation	Main Results	Reference
**Animal studies:**					
6-week-old male ICR mice 3 groups n = 8/group	Animal study	Forelimb grip strength Forced swim-to-exhaustion test, with loads 15-min swim test to determine recovery and fatigue-related biomarkers	*L. plantarum* TWK10 (LP10) Dosing per group: 0, 2.05 × 10^8^; or 1.03 × 10^9^ CFU/kg/day for 6 weeks	PRO improved forelimb grip strength and exhaustive swimming time. Blood lactate, ammonia, and CK levels were lower in PRO mice after a 15-min swim compared with those in control mice. Type I muscle fiber type increased, and relative muscle weight increased in PRO mice vs. control mice.	[88]
6-week-old male ICR mice 4 groups n = 8/group	Animal study	Forelimb grip strength Forced swim-to-exhaustion test with loads 10-min and 90-min swim tests, to determine recovery and fatigue-related biomarkers	A kefir drink with *L. fermentum* DSM 32,784 (LF26), *L. helveticus* DSM 32,787 (LH43), *L. paracasei* DSM 32,785 (LPC12), *L. rhamnosus* DSM 32,786 (LRH10), and *S. thermophilus* DSM 32,788 (ST30) Kefir dosing per group: 0, 2.15, 4.31, or 10.76 g/kg/day for 4 weeks	Kefir supplementation increased time-to exhaustion, and improved forelimb grip strength. Blood lactate, ammonia, blood urea nitrogen, and CK levels were lower after exercise in kefir-fed mice compared with control mice, in a dose-dependent manner. Glycogen contents in the liver and muscle were higher in kefir-supplemented mice compared with control mice.	[89]
11-week-old male Wistar rats 2 groups n = 13/group	Animal study	Incremental speed exercise on a treadmill, until exhaustion Treadmill chamber, coupled with gas-analyzer, to assess VO_2max_	*Saccharomyces boulardii* (strain not reported) 3 × 10^8^ CFU/kg/day for 10 days	PRO supplementation moderately improved aerobic performance. PRO mice ran approx. 8 min longer than control mice (until exhaustion) and had higher maximal speed.	[90]
7-week-old male ICR mice 4 groups n = 10/group	Animal study	Forelimb grip strength Forced swim-to-exhaustion test, with loads 10-min and 90-min swim tests, to determine recovery and fatigue-related biomarkers	*B. longum* subsp. *longum* OLP-01 isolated from a female weightlifter Dosing per group: 0, 2.05 × 10^9^, 4.10 × 10^9^, or 1.03 × 10^10^ CFU/kg/day for 4 weeks	PRO improved forelimb grip strength and swim-to-exhaustion time, in a dose-dependent manner. Blood lactate and ammonia levels were lower after the acute swim test in PRO vs. control mice. After a 90-min swim test, blood urea nitrogen and CK levels were lower in PRO mice compared with those in control mice. PRO increased hepatic and muscular glycogen contents, observed at autopsy.	[98]
6-week-old male ICR mice 4 groups n = 10/group	Animal study	Forelimb grip strength Forced swim-to-exhaustion test, with loads 10-min and 90-min swim tests, to determine recovery and fatigue-related biomarkers	*L. salivarius* subsp. *salicinius* SA-03, isolated from a female weightlifter’s gut microbiota Dosing per group: 0, 2.05 × 10^9^, 4.10 × 10^9^, or 1.03 × 10^10^ CFU/kg/day for 4 weeks	PRO improved forelimb grip strength and swim-to-exhaustion time, in a dose-dependent manner. Blood lactate and ammonia levels were lower and blood glucose levels were higher after acute tests in the PRO groups vs. control group. After a 90-min swim, blood CK levels were lower in PRO groups compared to the control group. PRO increased hepatic and muscular glycogen contents, observed at autopsy.	[99]
**Clinical studies:**					
**Swimmers**					
Highly trained competitive swimmers n = 17, females Age not reported	Randomized, double-blind, placebo-controlled	6 weeks of intensified off-season training, including swimming and resistance exercise. **Performance assessment**: Vertical jump force plate test, aerobic and anaerobic swim performance test **Cognitive assessment**: stress and recovery during the intensified exercise training load (the Recovery-Stress Questionnaire for Athletes)	*B. longum* 35,624; 1 × 10^9^ CFU bacteria/day for 6 weeks	No significant differences in exercise performance or systemic inflammation markers (at rest) between PRO and PLA. Differences in cognitive outcomes were detected showing more favorable sport recovery related scores in the PRO group.	[93]
Swimmers n = 46, females Age 13.8 ± 1.8 y	Randomized, placebo-controlled	Normal exercise regimen **Performance assessment:** 400-m free- swimming record, Harvard step test to, measure VO_2max_	*L. acidophilus SPP, L. delbrueckii* subsp. *bulgaricus, B. bifidum*, and *S. salivarus* subsp. thermophilus, strains not reported 400 mL of probiotic yogurt/day with 4 × 10^10^ CFU/mL for 8 weeks	Significant improvement in VO_2max_ in the PRO group. No differences in 400-m swimming times between PRO and PLA groups.	[82]
**Endurance runners**					
Elite distance runners n = 20, males Age 27.3 ± 6.4 y	Randomized, double-blind, placebo-controlled, crossover	Habitual winter-season training **Performance assessment**: A treadmill running test until exhaustion, at the start of the study period and the end of each study month	*L. fermentum* VRI-003; 1.2 × 10^10^ CFU bacteria/day for 4 weeks Cross-over study, with 1-month wash-out	No difference in performance outcomes with PRO compared to PLA. The number of illness days during PRO supplementation was significantly lower than with PLA (30 vs. 72 days). IFN-γ response was moderately higher with the PRO than with PLA.	[81]
Endurance-trained runners n = 8, males Age 26 ± 6 y	Randomized, blinded, placebo-controlled, cross-over	Habitual training **Bout of exercise**: 2-h running exercise at 60% VO_2max_ in hot ambient conditions	*L. casei* (strain not reported) 1 × 10^11^ CFU/day for 7 days Cross-over study, with 1-month wash-out	No differences in hydration status between PRO and PLA. Inflammatory cytokine levels were not different between PRO and PLA, either pre-exercise or post-exercise (1, 2, 4, and 24 h after running).	[100]
Endurance-trained runners n = 8, males Age 26 ± 6 y	Randomized, blinded, placebo-controlled, cross-over	Habitual training **Bout of exercise**: 2-h running exercise, at 60% VO_2max,_ in hot, ambient conditions	*L. casei* (strain not reported) 1 × 10^11^ CFU/day for 7 days Cross-over study with 1-month wash-out	PRO and PLA did not differ in salivary anti-microbial protein or serum cortisol responses during the post-exercise period (1, 2, 4, and 24 h after running).	[101]
Runners n = 10, males Age 27 ± 2 y	Randomized, double-blind, placebo-controlled, cross-over	Normal training **Performance assessment**: Running to fatigue, at 80% of ventilatory threshold, at 35 °C and 40% humidity	Multispecies probiotic, strains not specified; *L. acidophilus*, *L. rhamnosus, L. casei, L. plantarum, L. fermentum, B. lactis, B. breve, B. bifidum,* and *S. thermophilus* 45 × 10^9^ CFU/day for 4 weeks, cross-over study with a 3-week wash-out	PRO increased run time to fatigue (PRO 37:44 vs. PLA 33:00 min:sec). A moderate, non-significant reduction in pre-exercise and post-exercise serum lipopolysaccharide (LPS) levels for PRO compared to PLA. No difference between PRO and PLA in plasma IL-6, IL-10, and IL-1Ra or GI permeability after exercise in the heat.	[77]
Marathon runners n = 42, males Age 39.5 ± 9.4 y	Randomized, double-blind, placebo-controlled	Usual training **Bout of exercise**: marathon run	*L. casei* Shirota 40 × 10^9^ CFU/day for 30 days	PRO maintained salivary immune protection and increased anti-inflammatory response on the upper airways, immediately after the marathon. Serum TNF-α level was significantly lower immediately post-marathon in the PRO group compared to that in the PLA group	[102]
Marathon runners n = 119 (105/M, 14/F) Average age 40 y	Randomized, double-blind, placebo-controlled	3-month training period, 6-day preparation period **Bout of exercise**: marathon run	*L. rhamnosus* GG 4.0 × 10^10^ bacteria in drink/day (or 1 × 10^10^ in tablet/day) for 3 months	PRO did not differ from PLA in ox-LDL or antioxidant activity, pre- or post-marathon.	[103]
Marathon runners n = 24 (20/M, 4/F) Age 22–50 y	Randomized, double-blind, placebo- controlled, matched-pairs	Habitual training routine **Performance assessment/Bout of exercise**: Marathon race (no baseline assessment)	*L. acidophilus* CUL60, *L. acidophilus* CUL21, *B. bifidum* CUL20, *and B. animalis* subsp. *lactis* CUL34 2.5 × 10^10^ CFU/day for 28 days	No difference in marathon times between PRO and PLA. During the final third of the race, the reduction in average relative speed was greater in PLA compared to PRO. GI symptoms were lower in PRO compared to PLA during the final third. No difference in post-race serum IL-6, IL-8, IL-10, and cortisol levels between groups.	[104]
Ultramarathon runners n = 32 (26/M, 6/F) Age 23–53 y	Randomized, controlled (single-blind for glutamine supplementation)	Training for a marathon, ultra-marathon race of 294 km **Performance assessment**: A graded exercise test, to maximal exhaustion, on a motorized treadmill, VO_2max_ test, pre-marathon, time-to-completion in ultra- marathon race	PRO: Multi-strain probiotic, daily dose 30 × 10^9^ CFU comprising of 10 × 10^9^ CFU *L. acidophilus* CUL-60 (NCIMB 30,157), 10 × 10^9^ CFU *L. acidophillus* CUL-21 (NCIMB 30,156), 9.5 × 10^9^ CFU *B. bifidum* CUL-20 (NCIMB 30,172), and 0.5 × 10^9^ CFU *B. animalis* subsp. *lactis* CUL-34 (NCIMB 30,153 + 55.8 g fructooligosaccharides PRO + glutamine: Daily dose 2 × 10^9^ CFU *L. acidophilus* CUL-60 (NCIMB 30,157), 2 × 10^9^ CFU *L. acidophilus* CUL-21 (NCIMB 30156), 5 × 10^7^ CFU *B. bifidum* CUL-20 (NCIMB 30,172), 9.5 × 10^8^ CFU *B. animalis* subsp. *lactis* CUL-34 (NCIMB 30,153), and 5x 10^9^ CFU *L. salivarius* CUL61 (NCIMB 30,211) + 0.9 g glutamine 12 weeks before the marathon	No difference in pre-race VO_2max_ or in time-to-completion for ultra-marathon between PRO, PRO + glutamine, and control groups. PRO and PRO + glutamine had no effects on immune activation via extracellular heat-shock protein eHsp72 signaling at post-race.	[94]
**Cyclists, triathletes**					
Competitive cyclists n = 99 (64/M, 35/F) Age 35 ± 9 y/M and 36 ± 9 y/F	Randomized, double-blind, placebo-controlled	Habitual training (physical activity recorded) **Performance assessment**: an incremental cycle ergometer performance test (peak power output, VO_2max_)	*L. fermentum* VRI-003 PC 1 × 10^9^ CFU/day for 11 weeks	PRO did not affect training patterns or performance in VO_2 max_ testing. Acute exercise-induced changes in anti- and pro-inflammatory cytokines were attenuated with PRO.	[71]
Triathletes Study I: n = 18, Study II: n = 16 Sex not reported Age 19–26 y	Randomized, double-blind, placebo-controlled	8 weeks of programmed training before a sprint triathlon (Study I) or full triathlon competition (Study II) **Performance assessment**: Wingate and 85% VO_2max_ test (after full triathlon)	*L. plantarum* PS128 3 × 10^10^ CFU/day Study I: last 4 weeks of training Study II: last 3 weeks of training	In Study II, performance during recovery from a full triathlon was decreased in the PLA group and maintained at the pre-triathlon level in the PRO group. PRO group had lower blood TNF-α, IFN-γ, IL-6, and IL-8 levels compared to PLA, immediately after exercise (Study I/II), with levels significantly lower in PRO group 3 h after full triathlon (Study II). Anti-inflammatory IL-10 was higher in the PRO group, immediately after exercise (Study II) compared with that in the PLA group. No differences in muscle damage or fatigue markers detected between groups (Study I/II) except, lower CK in PRO vs. PLA, 3 h after full triathlon (Study II). Oxidative stress marker (MPO) was lower in PRO after exercise, with no differences 3 h post-exercise.	[105]
Elite athletes (badminton, triathlon, cycling, alpinism, karate, savate, kayak, judo, tennis, and swimming) n = 50 (36/M, 14/F) Age 18–28 y	Randomized, double-blind, placebo-controlled	Habitual training >11 h/week, self-reported training loads **Performance assessment**: VO_2max_, by a graded cardiopulmonary test, on a treadmill **Cognitive assessment**: Profile of mood and state (POMS) questionnaire	*L. helveticus* Lafti L10 2 × 10^10^ CFU/day for 14 weeks	No difference in VO_2max_ and treadmill performance between PRO and PLA. Increase in the subjective feeling of vigor in the PRO group, but no difference in other cognitive scores between groups.	[84]
Recreational triathletes n = 30 (25/M, 5/F) Age 35 ± 1 y	Randomized, double-blind, placebo-controlled	Standardized training program for the previous 6 months **Performance assessment/Bout of exercise**: a long-distance triathlon (no baseline assessment)	Multistrain probiotic, daily dose 30 × 10^9^ CFU (10 × 10^9^ CFU *L. acidophilus* CUL-60 (NCIMB 30,157), 10 × 10^9^ CFU *L. acidophillus* CUL-21 (NCIMB 30,156), 9.5 × 10^9^ CFU *B. bifidum* CUL-20 (NCIMB 30,172), 0.5 × 10^9^ CFU *B. animalis* subsp. *lactis* CUL-34 (NCIMB 30,153)) + 55.8 g fructo-oligosaccharides, alone or in combination with 600 mg *N*-acetyl carnitine + 400 mg α-lipoic acid for 12 weeks before and 6 days after triathlon	Non-significantly faster times were reported for PRO during swim and cycle stages, and a trend towards an overall faster time was reported compared to PLA (~86 min faster). No baseline measurements on performance were assessed. PRO reduced post-race plasma endotoxin levels, whereas PLA had no effect.	[73]
**Team sports**					
Division I volleyball and soccer athletes n = 23, females Age 19.6 ± 1.0 y	Randomized, double-blind, placebo-controlled	Offseason resistance training protocol **Performance assessment**: 1RM testing (bench press, squat, deadlift), isometric midthigh pull, vertical jump height, pro-agility test	*Bacillus subtilis* DE111 5 × 10^9^ CFU/day for 10 weeks	PRO had no effect on strength or athletic performance but significantly reduced percentage of body fat percentage.	[96]
Division I baseball athletes n = 25, males Age 20.1 ± 1.5 y	Randomized, double-blind, placebo-controlled	Resistance training program **Performance assessment**: 1RM testing (squat, deadlift), pro-agility test, 10-yard sprint, standing long jump	*Bacillus subtilis* DE111 1 × 10^9^ CFU/day for 12 weeks	No differences between PRO and PLA in strength, performance, or body composition. PRO reduced TNF-α levels, but no differences in IL-10, cortisol, zonulin, or testosterone levels observed between PRO and PLA.	[95]
Highly trained athletes n = 29 (13/M, 16/F) Age 20–35 years	Randomized, double-blind, placebo-controlled	Normal training **Performance assessment**: Cycle ergometer exercise test until exhaustion	*B. bifidum* W23, *B. lactis* W51, *Enterococcus faecium* W54, *L*. *acidophilus* W22, *L. brevis* W63, and *L. lactis* W58 1 × 10^10^ CFU/day for 12 weeks	No difference in performance between groups. Weekly training loads were significantly higher in PRO compared to PLA (8.0 ± 2.3 vs. 6.6. ± 4.3 h/week). Exercise-induced reduction in tryptophan levels in PLA but not in the PRO group. PRO reduced the incidence of URT infections.	[85]
**Active non-athletes**					
Resistance trained subjects n = 15, males Age 25 ± 4 y	Randomized, double-blind, placebo-controlled, crossover	Muscle-damaging eccentric exercise bout **Performance assessment**: isometric peak torque, after muscle damaging-exercise	*S. thermophilus* FP4, and *B. breve* BR03 5 × 10^9^ CFU of each/day for 21 days	PRO attenuated performance decrements caused by muscle-damaging exercise during the recovery period. No effects of PRO on muscle soreness, range of motion, or plasma creatine kinase. PRO lowered resting IL-6 concentrations that were sustained until 48 h post-exercise.	[106]
Recreational exercisers n = 29, males Age 21.5 ± 2.8 y	Single-blind, crossover (casein first, after washout, PRO+casein)	Single-leg exercise bout **Performance assessment**: Anaerobic power by modified Wingate test, single-leg vertical jump, strength, by 1RM testing in the one-legged leg press, after muscle damaging-exercise	*Bacillus coagulans* BC30 1 × 10^9^ CFU/day + 20 g casein for 14 days	PRO + casein increased perceived recovery status and reduced muscle soreness after exercise compared with casein alone. PRO + casein maintained post-exercise Wingate peak power at the pre-exercise level, whereas casein alone demonstrated reduced post-exercise performance. For 1RM leg-press and vertical jump power, no differences between groups in post-exercise performance.	[107]
Physically active subjects n = 27, females Age 18–25 y	Controlled, randomized	Habitual moderate exercise **Performance assessment**: treadmill running until exhaustion, VO_2max_ test (Bruce test)	Probiotic not specified 450 g of probiotic yogurt/day for 2 weeks	No difference in VO_2max_ between PRO and PLA. PRO yogurt increased antioxidant enzyme activities and reduced MMP2 and MMP9 levels before and after exhaustive exercise. No significant differences between PRO and PLA in high-sensitivity CRP, IL-6, and TNF-α after intense exercise.	[108]
Physically active students n = 11, sex not reported Age 22 ± 1 y	Non-controlled	Habitual training including endurance exercise **Bout of exercise**: 2-h cycling at 60% of VO_2max_	*L. acidophilus, L. delbrueckii* subsp. *bulgaricus, Lactococcus lactis* subsp. *lactis*, *L. casei, L. helveticus, L. plantarum, L. rhamnosus, L. salivarius* subsp. *salivarius, B. breve, B. bifidum, B. infantis, B. longum, Bacillus subtilis, S. thermophilus* minimum 2 × 10^9^ CFU/capsule, 3 capsules/day for 30 days	Rating of perceived exertion during exercise was not different between PRO and PLA. PRO did not affect salivary antimicrobial proteins at rest or in response to an acute bout of prolonged exercise.	[109]
Students n = 67, males and females (n not specified by sex) Age 18–24 y	Controlled	The exercise groups completed structured, long-distance, endurance run training, whereas the active group maintained their usual exercise routine. **Performance assessment**: 1.5-mile (2.41 km) walk or run	Probiotic kefir, probiotic strain and dose not specified 15 weeks	No effect of PRO on 1.5-mile completion time. PRO attenuated exercise-induced inflammation, measured as serum CRP levels.	[110]
Students of physical education n = 30, males Average age: PRO 21.56 y, PLA 21.28 y	Randomized, matched pairs	Habitual training and training program by the study **Performance assessment**: Cooper test, maximum aerobic power, using Bulk test on a laboratory treadmill	Probiotic strains unspecified, included *S. thermophilus* and/or *L. delbrueckii* subsp. *bulgaricus* 1 × 10^5^ CFU/g in 200 mL yogurt/day for 10 weeks	PRO improved VO_2max_ and aerobic performance. PRO decreased serum high-sensitivity CRP and increased HDL levels.	[111]
Healthy participants n = 16, males Age 20–40 y	Randomized, double-blind, placebo-controlled	Habitual exercise **Performance assessment**: Treadmill running at 85% VO_2max_ workload, until exhaustion.	*L. plantarum* TWK10 1 × 10^11^ CFU/day for 6 weeks	PRO improved time-to-exhaustion (PLA vs. PRO: 817 ± 79 s vs. 1292 ± 204 s). Blood glucose was higher in PRO vs. PLA after exhaustive exercise. No differences in post-exercise blood lactate, free fatty acid, CK levels between PRO and PLA.	[91]
Healthy participants n = 54, (27/M, 27/F) Age 20–30 y	Double-blind, placebo-controlled	Habitual exercise **Performance assessment**: treadmill running, at 60% VO_2max_ and 85% VO_2max_ workload, until exhaustion	*L. plantarum* TWK10 3 × 10^10^ CFU/day or 9 × 10^10^ CFU/day for 6 weeks	Exhaustion time was increased in both PRO groups and were longer compared to PLA. Improvement in exercise capacity was dose-dependent. PRO reduced serum lactate during and after exercise compared to PLA. Muscle mass increased in the high-dose PRO group.	[92]
Healthy sedentary individuals n = 41, males Age 19–26 y	Randomized, parallel, placebo-controlled	Circuit training protocol, including resistance exercises, 3 times a week **Performance assessment**: muscular strength (peak torque) and power via an isokinetic dynamometer	*L. acidophilus* BCMC 12,130, *L. casei* BCMC 12,313, *L. lactis* BCMC 12,451, *B. bifidum* BCMC 02,290, *B. infantis* BCMC 02,129 and *B. longum* BCMC 02,120 6 × 10^10^ CFU/day for 12 weeks	PRO did not show superior effects to PLA on muscular strength (peak torque) and power. PRO alone and exercise alone increased post-intervention serum IL-10 concentrations from pre-intervention levels. PRO and PLA with or without exercise, had no effects on serum IL-6 concentration.	[97]
Healthy elderly individuals with stretching experience n = 29 (14/M, 25/F) Age > 65 y	Randomized, double-blind, placebo-controlled	Moderate resistance exercise training, in instructed classes and at home **Cognitive assessment**: General cognitive performance (incl. tests for accuracy, reaction time), mental state (scoring for depression, anxiety, and overall mental state)	*B. longum* BB536, *B. infantis* M-63, *B. breve* M-16V *and B. breve* B-3 5 × 10^10^ CFU/day (1.25 × 10^10^ CFU each probiotic/day) for 12 weeks	An increase in the general cognitive function scores was observed in PRO and PLA groups, at 12 weeks. PRO group showed a decrease in anxiety-depression scores, body weight, BMI and body fat.	[112]

ICR mice, Institute of Cancer Research mice; *L., Lactobacillus* (or related genera)*;* *B., Bifidobacterium; S., Streptococcus;* CFU, colony-forming units; PRO, probiotic supplementation; PLA, placebo supplementation; CK, creatine kinase; VO_2max_, maximum rate of oxygen consumption; M, males, F, females; IFN-γ, interferon γ; IL, interleukin; GI, gastrointestinal; TNF-α = tumor necrosis factor α; ox-LDL, oxidized low-density lipoprotein; MPO, myeloperoxidase; 1RM, 1 repetition maximum; MMP2/9, matrix metalloproteinase 2/9; CRP, C-reactive protein; HDL, high-density lipoprotein; BMI, body mass index.

Thus far, the number of human clinical studies investigating the impacts of probiotics on physical performance remains low, and those that have been performed have examined limited exercise types and performance measures. Clinical data have suggested that probiotics may improve the time to exhaustion during endurance exercise; however, these data are scarce and contradictory results exist. Studies have been conducted using a variety of probiotic strains that may differ in their efficacy. Further research remains necessary to determine the direct effects of probiotic supplementation on performance outcomes.

### 3.3. Improvement in Post-Exercise Recovery

Recovery from exercise represents an important determinant of performance enhancement that enables adaptation to training. Strategies for optimizing recovery may prevent under-recovery, overtraining syndrome, injuries, or illnesses [86]. Exercise-induced muscle damage, inflammation, metabolic responses, and fatigue are part of the recovery process and are, therefore, important contributors to training adaptation. In addition to physical performance outcomes, biochemical markers and the athlete’s subjective perception of fatigue and readiness to perform can be assessed, to evaluate the subject’s recovery state after exercise.

The impacts of probiotic supplementation on health outcomes, performance measurements, and/or biochemical markers in athletes have been addressed in numerous studies, comparing post-intervention and pre-intervention resting levels after a training period. Endpoints and markers that are measured directly after acute exercise sessions and during recovery periods provide a more defined approach to the evaluation of post-exercise recovery status. The effects of probiotics on biochemical and immune markers during the post-exercise recovery state after an exercise session have been reported in several studies (Table 2). The effects of probiotic supplementation on performance capacity during the recovery period after exercise were studied in triathletes by Huang et al. [105], who assessed anaerobic (Wingate test) and aerobic (85% VO_2max_ test) exercise capacities, 48 and 72 h after a triathlon race. Probiotic supplementation (*L. plantarum* PS128) for 3 weeks significantly improved maximal power, the fatigue index, and endurance indices during the recovery period after the triathlon race compared with those in the placebo group. The probiotic group also maintained aerobic performance, when measured during the recovery period at the resting level, whereas the placebo group reached exhaustion significantly sooner during the recovery period

High-intensity training acutely increases muscle damage, fatigue, and soreness, which contributed to decreased athletic performance. Excess mechanical load creates micro-damage to skeletal muscle tissues, causing local inflammation and decreasing muscle function. Inflammation that occurs in the muscle tissue is a mechanism of muscular adaptation to exercise, through which the muscle can regenerate and repair itself [113]. Mechanical overload has been associated with increased systemic levels of muscle-derived proteins, such as creatine kinase (CK) and myoglobin [107]. Interleukin (IL)-6 is a cytokine that is produced by contracting muscles during exercise and increases in the plasma after strenuous exercise. Changes in muscle-damage-related biomarkers are associated with delayed-onset muscle soreness (DOMS) and muscle recovery [114].

In athletes who participated in a full triathlon championship competition, the *L. plantarum* PS128 probiotic group and the placebo group did not differ in blood CK values immediately after competition [105]. However, in the probiotic group, the CK level was significantly lower 3 h post-exercise compared with that in the placebo group. No differences in post-exercise lactate dehydrogenase, myoglobin, or free fatty acids were observed between the probiotic and placebo groups. After less-demanding sprint triathlon, supplementation with *L. plantarum* PS128 had no effects compared with placebo on post-exercise CK or blood lactate measurements [105]. In sedentary subjects who participated in exhaustive exercise, *L. plantarum* TWK10 improved blood lactate clearance during a 1-h post-exercise recovery period [92]. Blood lactate and lactate clearance are often measured to assess recovery. However, the suitability of these variables to evaluate fatigue and recovery is controversial and not agreed on [86].

In the triathlete study performed by Huang et al. [105], the levels of exercise-induced serum pro-inflammatory cytokines, tumor necrosis factor (TNF)-α, interferon (IFN)-γ, IL-6, and IL-8, were significantly lower in the probiotic group compared with those in the placebo group, both immediately and 3 h after the triathlon competition. The investigators also found increased anti-inflammatory IL-10 levels after the exercise, but not at the 3-h time point. Prolonged, high-intensity exercise is well-known to be associated with transient inflammation, immune dysfunction, and oxidative stress [78]. Lamprecht et al. [72] and Mazani et al. [108] both demonstrated a trend towards reduced circulating TNF-α levels in the probiotic group compared with the placebo, immediately after exhaustive exercise, supporting the findings reported by Huang et al. [105]. Probiotic interventions were found to increase antioxidant capacity [108], reduce oxidated molecules [72], and decrease myeloperoxidase and increase thioredoxin activity [105], suggesting overall benefits associated with reduced exercise-induced oxidative stress levels. However, some probiotic intervention studies have not found any effects on inflammation [93,100,101,104]; thus, further investigations are warranted to understand the effects of probiotics on post-exercise immune function and inflammation.

Increased levels of inflammatory cytokines may result from damaged muscle tissue but may also be caused by the disruption of intestinal barrier function after prolonged, intense, endurance exercise. Reduced intestinal blood flow causes the acute disruption of epithelial barrier function and increased leakage, resulting in endotoxemia, during which microbial lipopolysaccharides enter the blood circulation. The resulting systemic inflammation compromises the athlete’s ability to recover and perform. Lamprecht et al. [72] showed that a 14-week, multi-strain, probiotic supplementation protocol reduced fecal zonulin and TNF-α levels significantly compared with those supplemented with placebo, indicating improved intestinal barrier integrity and reduced systemic inflammation, respectively. Probiotic supplementation of shorter duration for 4 weeks resulted in reduced gastrointestinal permeability and improved exercise capacity under heat conditions, with no impacts on circulating cytokine levels [77]. Moreover, probiotic supplementation did not attenuate exertional heat stress-induced blood endotoxemia or inflammation [100], the salivary antimicrobial protein response [101], or extracellular heat shock protein 72 (eHsp72) concentrations [94], when monitored during the recovery stage, post-exercise. Under normal ambient conditions, a 30-day supplementation protocol using a multi-strain probiotic did not demonstrate differences in the salivary antimicrobial peptides during post-exercise recovery after 2 h of cycling at 60% of VO_2max_ [109].

The benefits of probiotic use during recovery from muscle-damaging exercise have been demonstrated in two clinical studies [106,107]. A study performed in resistance-trained men, demonstrated that a 3-week supplementation with *S. thermophilus* FP4 and *B. breve* BR03 moderately attenuated post-exercise decreases in muscle performance, as assessed by isometric average peak torque, 24 to 72 h after a muscle-damaging exercise [106]. In addition, circulating IL-6 concentrations were reduced after the 3-week probiotic supplementation protocol but were not affected by the treatment during the post-exercise recovery period. Beneficial effects were observed in the resting arm angle after probiotic supplementation, whereas no differences in flexed arm angle, CK levels, or muscle soreness were observed during the recovery period, between the probiotic and placebo groups.

A 2-week supplementation of casein combined with *Bacillus coagulans* BC30, increased perceived recovery status scores at 24 and 72 h after muscle-damaging exercise compared with casein supplementation alone in recreationally trained men [107]. Probiotic combined with casein also reduced perceived muscle soreness compared with casein alone, 72 h post-exercise. Trends toward reduced circulating CK levels and improved performance, as measured by the Wingate test, were observed after the muscle-damaging exercise following probiotics combined with casein supplementation compared with casein supplementation alone. The amounts of muscle swelling and blood urea nitrogen levels did not differ between the groups. The effects of *B. coagulans* BC30 have also been studied among soldiers, who are known to train intensively, on a daily basis, with limited time to recover. β-Hydroxy-β-methylbutyrate calcium (CaHMB) combined with BC30 maintained muscle integrity during an intensive 40-day military training period better than CaHMB alone [115]. Treatment with both CaHMB combined with BC30 and CaHMB alone significantly attenuated resting serum IL-1β, IL-2, and TNF-α concentrations after the 40-day supplementation period, whereas CaHMB that was combined with BC30 significantly reduced serum IL-6 and IL-10 during the post-intervention period compared with control. However, the acute effects on biochemical marker levels during the recovery state were not evaluated. Probiotics have been proposed to enhance recovery and to shorten the time necessary for muscle repair by improving the absorption and utilization of dietary nutrients [107,115,116].

To date, studies that have assessed performance and exercise capacity during the post-exercise recovery period remain low in number. Studies that have investigated the probiotic effects on biochemical and immune markers during the post-exercise recovery period have shown somewhat controversial results, due to large variations in study designs, training protocols, analytical methods, athletic populations, and investigated probiotic strains. These results also warrant longer follow-up measurements during the recovery period. Thus, conclusions cannot be drawn regarding probiotics’ potential to improve recovery and attenuate exercise-induced physiological responses, which are, in part, necessary for training adaptations and performance enhancement. Furthermore, the relationships between physiological recovery processes and improvements in performance should be established more clearly before further conclusions can be made regarding the ergogenic potential of probiotics.

### 3.4. Improvements in Mood-Related Outcomes

Good physical condition, accompanied by good mental condition, are part of a continuum that enables the optimal training and performance of competitive athletes. Fatigue and mood disturbances during performance are common among athletes during the training season and in competition [4]. Intensive exercise causes both physical and psychological stress responses, which can often be difficult to differentiate between.

Results from preclinical and clinical studies suggest that probiotic administration may have positive effects in mental responses [117,118]. Few studies have investigated the effects of probiotic supplementation on the cognitive outcomes of athletes or physically active subjects [84,93,112] (Table 2). In a group of highly trained, elite athletes, the self-rated sense of vigor (Profile of Mood States, POMS questionnaire) was significantly increased among the probiotic group, who ingested *L. helveticus* Lafti for 14 weeks, compared with the placebo group, with no difference in the total mood disturbance scores between groups detected [84]. Decreased vigor is related to an individual’s feelings of possessing the necessary physical strength to perform.

In a study involving highly trained, female, competitive swimmers, probiotic supplementation (*B. longum* 35,624) during a 6-week intensive training period improved the cognitive functions of the athletes [93]. At the end of the intensive training period, significant differences in the scores related to sport recovery categories (the Recovery-Stress Questionnaire for Athletes) were detected between groups, showing that the scores of the probiotic group were more favorable compared with those in the placebo group. A training intervention performed in healthy, elderly, Japanese individuals demonstrated that a 12-week resistance training program induced beneficial effects on the general cognitive functions of both the placebo and probiotic groups [112]. The 12-week supplementation with multi-strain bifidobacteria significantly decreased overall mental state scores compared with baseline scores, with lower scores indicating lower depression and anxiety symptoms [112].

Probiotics are, by definition, live microorganisms. In addition to viable bacteria, studies have been performed using inactivated bacteria. Two studies have investigated the effects of supplementation with inactivated *Lactobacillus* on mood related measurements [119,120]. A 4-week supplementation period, using heat-inactivated *L. gasseri* OLL2809, reduced tension-anxiety scores after a 1-h cycle ergometer exercise, compared with baseline scores [119]. The 12-week administration of heat-inactivated *L. gasseri* CP2305 significantly decreased scores that measured physical fatigue, anxiety, and depression in male university student-athletes [120]. Salivary cortisol and chromogranin A serve as biochemical markers for stress. In the above-mentioned studies, salivary chromogranin A was significantly reduced in the inactivated *Lactobacillus* group compared with that in the placebo group [120], whereas no changes in salivary cortisol levels were detected after the intervention period [119,120].

Probiotics appear to have benefits on cognitive outcomes in athletes, as measured by self-reported scores. Several potential mechanisms exist for the gut bacteria to interact with the brain, through the gut-brain axis. Messages to the brain can be delivered by gut-derived cytokines, hormones, and bacterial metabolites, including neurotransmitters, or via the vagus nerve [4]. Probiotic studies that focus on the mental health of athletes represent an emerging area in the field of sports nutrition and exercise performance. The number of probiotic studies remains very low, with studies often including a low number of subjects, and a wide variety of questionnaires have been used to assess cognitive outcomes. Despite limited evidence, cognitive health remains an intriguing area of sports nutrition research.

## 4. Conclusions

Overall, growing evidence from animal and human studies has indicated that the gut microbiota composition plays an important role in host physiology and can affect physical performance. The microbial community of the gut and its potential health benefits are highly impacted by individual life choices, including dietary patterns and activity levels. Probiotics are known for their potential to reduce GI and URT symptoms and infection episodes and thus may benefit the athlete by increasing the numbers of healthy training days and completed races. Further, probiotics may support athletic performance by enhancing training adaptations, attenuating physiological responses during post-exercise recovery periods, and improving mood and mental responses after intense exercise. Therefore, probiotics can be considered to act as indirect ergogenic aids; however, the causal impacts of indirect effects on performance remain to be established in good-quality, long-term studies of adequate size that consider the diet, and the training and competition seasons of the athletes. The functions of probiotics in enhancing performance, as direct ergogenic aids, require additional research that targets the mode of action that underlies their potential benefits.
